# Bone marrow macrophages support prostate cancer growth in bone

**DOI:** 10.18632/oncotarget.6042

**Published:** 2015-10-08

**Authors:** Fabiana N. Soki, Sun Wook Cho, Yeo Won Kim, Jacqueline D. Jones, Serk In Park, Amy J. Koh, Payam Entezami, Stephanie Daignault-Newton, Kenneth J. Pienta, Hernan Roca, Laurie K. McCauley

**Affiliations:** ^1^ Department of Periodontics and Oral Medicine, University of Michigan School of Dentistry, Ann Arbor, MI, USA; ^2^ Department of Internal Medicine, National Medical Center, Jung-gu, Seoul, Korea; ^3^ Department of Medicine, Vanderbilt University School of Medicine, Nashville, TN, USA; ^4^ Department of Biochemistry and Molecular Biology, Korea University College of Medicine, Seoul, Korea; ^5^ Center for Cancer Biostatistics, University of Michigan, Ann Arbor, MI, USA; ^6^ The James Buchanan Brady Urological Institute, Johns Hopkins University School of Medicine, Baltimore, MD, USA; ^7^ Department of Pathology, University of Michigan Medical School, Ann Arbor, MI, USA

**Keywords:** macrophages, prostate cancer, skeletal metastasis, MAFIA mouse, clodronate liposome

## Abstract

Resident macrophages in bone play important roles in bone remodeling, repair, and hematopoietic stem cell maintenance, yet their role in skeletal metastasis remains under investigated. The purpose of this study was to determine the role of macrophages in prostate cancer skeletal metastasis, using two *in vivo* mouse models of conditional macrophage depletion. RM-1 syngeneic tumor growth was analyzed in an inducible macrophage (CSF-1 receptor positive cells) ablation model (MAFIA mice). There was a significant reduction in tumor growth in the tibiae of macrophage-ablated mice, compared with control non-ablated mice. Similar results were observed when macrophage ablation was performed using liposome-encapsulated clodronate and human PC-3 prostate cancer cells where tumor-bearing long bones had increased numbers of tumor associated-macrophages. Although tumors were consistently smaller in macrophage-depleted mice, paradoxical results of macrophage depletion on bone were observed. Histomorphometric and micro-CT analyses demonstrated that clodronate-treated mice had increased bone volume, while MAFIA mice had reduced bone volume. These results suggest that the effect of macrophage depletion on tumor growth was independent of its effect on bone responses and that macrophages in bone may be more important to tumor growth than the bone itself. In conclusion, resident macrophages play a pivotal role in prostate cancer growth in bone.

## INTRODUCTION

The skeleton, a favored organ for metastasis, is associated with significant morbidity for cancer patients. In skeletal metastasis, the interplay of tumor cells with the bone microenvironment affects bone remodeling and tumor growth [1, 2]. Macrophages are myeloid phagocytic cells recruited in response to infection, inflammation and tissue injury, playing roles in the innate and adaptive immune response [3, 4]. They are activated differentially according to stimuli provided, M1 anti-tumorigenic macrophages are classically activated and M2 pro-tumorigenic macrophages (also known as tumor associated macrophages or TAMs) are alternatively activated [5, 6]. TAMs are prominently found and involved with cancer initiation, progression and metastasis, facilitating angiogenesis, matrix breakdown and tumor cell-motility [4, 5, 7, 8]. Clinical studies in prostate cancer demonstrated that overexpression of colony-stimulating factor (CSF-1) and its receptor (CSF1R or c-FMS), were responsible for monocyte and macrophage expansion, indicating poor prognosis in primary prostate cancers and development of bone metastasis [9]. Modification of myeloid cells in bone has demonstrated influence on tumor metastasis to bone. Prostate cancer cell localization and growth in bone was increased when a single dose of cyclophosphamide, a bone marrow-suppressive chemotherapeutic drug, was administered prior to intracardiac tumor inoculation [10]. This was associated with a transient expansion of myeloid cells and increased cytokines with myelogenic potential including C-C chemokine ligand 2 (CCL2), interleukin-6 (IL-6), and VEGF-A that primed the environment for tumor growth. Moreover, the chemokine CCL2 known to attract and differentiate macrophages towards TAMS and osteoclasts has been shown to increase prostate cancer growth and bone metastasis [11-15]. TAMs have been implicated in tumor growth, progression and metastasis of different types of cancer, but little is known about their role in skeletal metastasis.

Macrophages residing in bone, termed ‘osteomacs’ constitute one sixth of the total cells in the marrow and differ from osteoclasts, by expressing specific surface markers: F4/80 and CD68 [16, 17]. Interestingly, depletion of osteomacs compromises osteoblastic bone formation [3, 17]. Bone resident macrophages are found in close association with osteoblasts and are important in bone remodeling, bone healing and hematopoietic niche maintenance [3, 18, 19]. Moreover, osteomacs have important roles in skeletal homeostasis and PTH anabolic actions in bone where differences in bone remodeling were linked to the differentiation stages of the targeted macrophages [20]. Depletion of early lineage macrophages resulted in osteopenia whereas differentiated macrophage depletion presented the opposite effect of an osteogenic environment and enhanced PTH anabolism. These ‘osteomacs’ play important roles in the bone niche; however, their role in pathology such as skeletal metastasis remains elusive. Given the importance of macrophages in bone homeostasis, the purpose of this study was to determine the role of macrophages in prostate cancer metastatic growth in bone.

## RESULTS

### Macrophage ablation in MAFIA mice decreases prostate tumor growth in bone and total bone volume

**Figure 1:**
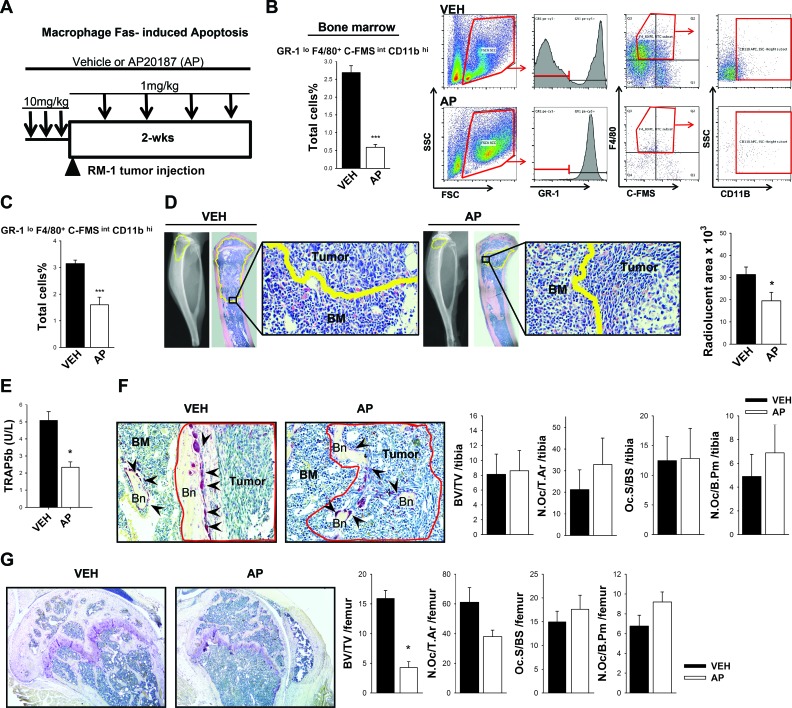
Macrophage ablation in MAFIA mice hindered tumor growth in bone and significantly decreased bone volume **A.** Macrophage depletion regimen and intratibial tumor growth. Sixteen week old male MAFIA mice were treated with AP20187 (AP) (10mg/kg) or vehicle control (VEH) for 3 days (black arrows). RM-1 murine prostate cancer cells were injected intratibially on the fourth day (black triangle) and booster injections were given every third day (1mg/kg) for depletion maintenance. Fourteen days later mice were euthanized and evaluated. **B.** Graph and representative flow cytometric plots of whole bone marrow cells after 3 days of initial macrophage depletion and quantitative analyses of GR-1^lo^ F4/80^+^ C-FMS^int^ SSC^int/lo^ CD11B^hi^ macrophages. **C.** FACS analyses of GR-1^lo^ F4/80^+^ C-FMS^int^ SSC^int/lo^ CD11B^hi^ macrophages in intratibial RM-1 tumors at day 14 from MAFIA mice treated with vehicle or the macrophage depleting AP compound. **D.** Radiographic and histological analyses of intratibial RM-1 tumors in vehicle (VEH) and AP20187 (AP) treated mice at day 14. Tumors are highlighted in yellow and 20X images show the edges of tumor and bone marrow cells (BM). Radiolucent areas were quantified in radiographic images of intratibial tumors shown in graph (mm^3^). **E.** Serum analyses of TRAP5b (units per liter) from vehicle (VEH) and AP20187 (AP) treated mice with intratibial RM-1 tumors at day 14. **F.** Histologic images (20X) of RM-1 tumors in MAFIA mice tibiae at day 14. Tumors within the marrow are delineated in red; bone (Bn), bone marrow (BM) tumor-free areas and TRAP positive osteoclasts (black arrows) are indicated. Bone histomorphometric analyses including bone volume (BV/TV), osteoclast numbers per total tissue area (N.Oc/T.Ar), osteoclast surface per bone surface (Oc.S/BS) and osteoclast numbers per bone perimeter (N.Oc/B. Pm) are shown. There was no difference in these histomorphometric parameters between vehicle and AP mice. **G.** Histomorphometric analyses at day 14 of the non-tumor containing femurs from the MAFIA mice bearing RM-1 tumors in the tibiae and treated with vehicle (VEH) or AP20187 (AP). Data for figures C-G are mean ±SEM, *n* = 6-7 per group. Data for figure B is mean ±SEM, *n* = 5 per group. Statistically significant differences were calculated using unpaired *t* tests between treatments. *P < 0.05, ***P < 0.001 vs. VEH.

The macrophage Fas-induced apoptosis (MAFIA) mouse provides for conditional macrophage depletion of cells that express GFP under the c-FMS promoter (CSF-1 receptor) [21]. The RM-1 metastatic murine prostate cancer cell line was used in this model, since it is compatible with the C57BL/6J background of the MAFIA immunocompetent mice. This is of noted importance, especially in studies with emphasis on the immune response. After 3 consecutive daily injections with the dimerizer AP20187 (AP) for initial macrophage depletion, RM-1 mouse metastatic prostate cancer cells [22] were inoculated in the proximal tibia, and tumors were monitored for 2 weeks (Figure 1A). The efficiency of macrophage (Gr1^lo^, F4/80^+^, c-FMS^int^ and CD11b^hi^) depletion was validated, before tumor inoculation (i.e. after 3 consecutive daily injections) (Figure 1B and Supplemental Figure 1).

Fourteen days after tumor inoculation into macrophage depleted mice, hind limbs were collected and tumor associated macrophage depletion was confirmed by FACS analyses (Figure 1C). Tumor sizes were analyzed by radiolucent area quantification and confirmed by histology (Figure 1D). Interestingly, macrophage-depleted mice had significantly reduced osteolysis in bone compared to vehicle-treated controls.

As previously reported, macrophage depletion highlights the important roles macrophages play in bone formation, healing, and PTH dependent anabolic actions [18, 20] and hence likely in skeletal tumor growth. Since macrophages share precursors with osteoclasts in bone, serum TRAP5b, a bone resorption marker, was analyzed (Figure 1E). Mice had significantly reduced levels of serum TRAP5b after 2 weeks of macrophage depletion compared to vehicle controls. Histomorphometric analyses were performed in tibiae with RM-1 tumors (Figure 1F) and showed no significant differences in total bone with macrophage ablation. To validate the effect of macrophage depletion in bone, histomorphometric analyses were also performed in the non-tumor containing femurs. Significant reductions in bone volume were observed in femurs of macrophage depleted mice confirming a macrophage depletion effect in bone homeostasis as previously reported (Figure 1G) [20]. Even though serum TRAP5b levels were significantly reduced with macrophage depletion, osteoclast number per tissue area (N.Oc/T.Ar), osteoclast number per bone perimeter (N.Oc/B.Pm), and osteoclast surface per bone surface (Oc.S/BS), were not significantly changed in tibiae or femurs with macrophage ablation.

To determine whether differences in bone volume observed in MAFIA AP-treated mice were due to a direct effect on osteoblast function, primary calvarial cells from MAFIA mice were isolated and treated with VEH or AP to induce macrophage depletion *in vitro* (Supplemental Figure 2). In a subset of cells after 1 day of treatment, flow cytometric analysis demonstrated a 32% decrease in the F4/80+ macrophage population (around 10% of cells were F4/80+ in the VEH group; Supplemental Figure 2A). After AP treatment, cells were then treated with ascorbic acid alone or ascorbic acid (AA) plus β-glycerol phosphate (BGP) for 12 and 14 days respectively for gene expression and mineralization analyses. The enrichment of the osteoblast population with the *in vitro* depletion of osteal macrophages resulted in increased mineralization (Von Kossa staining) and elevated osteocalcin gene expression (a marker of osteoblast differentiation) (Supplemental Figures 2B-2D). While these results do not definitely demonstrate a direct effect on osteoblasts, they suggest that the *in vitro* effect of AP in a culture of enriched osteoblasts is clearly not inhibitory. Therefore, *in vivo*, AP may alter osteoclast resorption activity and bone-remodeling homeostasis resulting in significant reduction of BV/TV found in non-tumor bearing femurs. However, in tumor-bearing tibiae, the absence of differences seen in the bone volume between VEH and AP groups appears not to be via direct effects in the osteoblast population. In this case, the results could be due to the predominant osteolytic activity induced by the RM-1 tumors, especially when tumors are larger in the VEH group (i.e. less bone), equalizing the differences in BV/TV observed in the tumor-free femurs. Overall, these data suggest that macrophage ablation in MAFIA mice hinders tumor growth in bone and significantly decreases total bone volume.

### Macrophage ablation in MAFIA mice hinders prostate cancer subcutaneous growth in an osseous implant model

**Figure 2:**
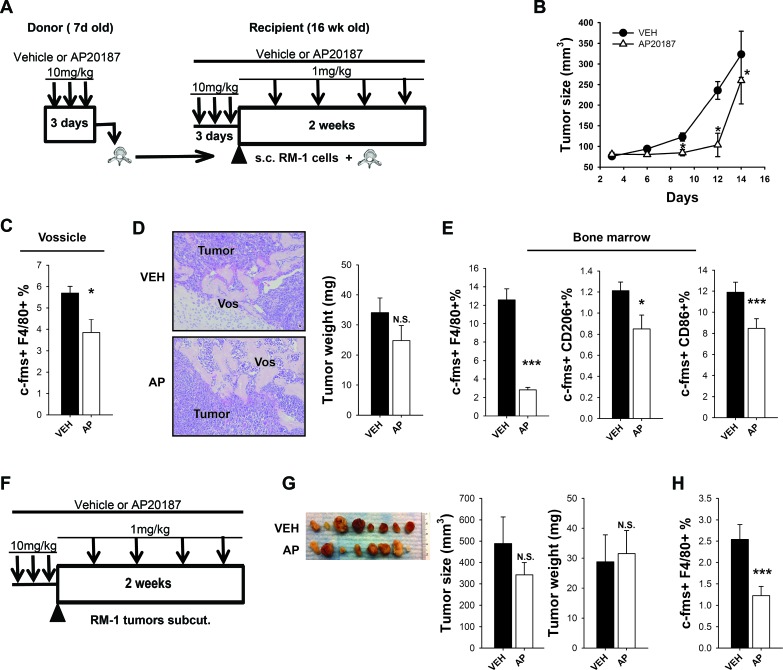
Macrophage ablation in MAFIA mice hindered prostate cancer growth in a bone microenvironment (vossicle model) **A.**-**E.** MAFIA mice with subcutaneous RM-1 tumors in vossicles. **A.** Experimental design for vossicle model with subcutaneous tumors. Sixteen week old MAFIA recipient mice (*n* = 6) and 7 day old donor mice (*n* = 9-11) were treated for 3 days with vehicle control (VEH) or AP20187 (AP). Vertebrae from donor mice were collected and implanted subcutaneously with RM-1 prostate cancer cells in the back of VEH (vertebrae from VEH-treated mice + VEH-treated recipients) or AP (vertebrae from AP-treated mice + AP-treated recipients) mice. Booster injections were given every third day (1mg/kg) for depletion maintenance and tumors were analyzed after 14 days (*n* = 7-9 per group). **B.** Vossicle tumor growth. Tumor sizes were measured by caliper every 3 days for 14 days. Tumors in macrophage ablated mice were significantly smaller than controls at day 9, 12 and 14. Data shows tumor size (average of 4 tumors per mouse). Statistical analysis for tumor growth was performed by repeated measures linear model, *P < 0.05 *vs*. VEH. **C.** Tumors in vossicles were analyzed by FACS for macrophage c-FMS+F4/80+% of total cells. **D.** Representative vossicle-tumor H&E sections showing the vossicle (Vos) surrounded by the tumor cells (20X). Tumor weight (mg) was measured 14 days after implantation. **E.** FACS analysis of macrophages in the whole bone marrow of tibiae shows macrophage depletion with AP treatment for macrophages (c-FMS+F4/80+), M2 macrophages (F4/80+CD206+) and M1 macrophages (F4/80+ CD86+). **F.**-**H.** MAFIA mice with subcutaneous RM-1 tumors in matrigel. **F.** Experimental design for subcutaneous RM-1 tumors in matrigel in MAFIA mice. Sixteen week old MAFIA mice (*n* = 8/group) were treated for 3 days with vehicle (VEH) or AP20187 (AP) for initial depletion. On the fourth day RM-1 prostate cancer cells mixed with growth factor-reduced Matrigel^TM^ were injected subcutaneously in the back of vehicle or AP treated mice (2 tumors/mouse). Booster injections were given every third day (1mg/kg) for depletion maintenance and tumors were analyzed after 14 days. **G.** Representative images of subcutaneous tumors of VEH or AP treated mice (1 tumor/mouse) and graphs for tumor size (mm^3^) and tumor weight (mg) (average of 2 tumors per mouse). **H.** FACS analyses for c-FMS+ F4/80+ macrophages in subcutaneous tumors. Data for figures B-E are mean ±SEM, *n* = 7-9 per group. Data for figures G and H are mean ±SEM, *n* = 5 per group.. Statistically significant differences were calculated using unpaired t tests between treatments for figures C-H. *P < 0.05, ***P < 0.001 *vs*. VEH, N.S. = non-significant.

To further investigate the impact of macrophages in prostate tumor growth in bone, murine RM-1 prostate cancer cells were injected in vertebral bodies (vossicles) [23] of MAFIA mice previously treated with vehicle or AP20187 for macrophage depletion, and subcutaneously implanted in the back of MAFIA mice hosts with or without initial macrophage depletion. To minimize any compensation of macrophages coming from the donor's vossicles, AP treated vossicles were implanted into AP-treated host mice, and vehicle treated vossicles were implanted in vehicle treated host mice. The rationale was to provide the highest and lowest macrophage initial pool for vossicle tumor growth. Tumors were then measured every 3 days for up to 2 weeks, and booster AP or vehicle injections were given every 3 days to maintain macrophage ablation (Figure 2A).

Statistical analyses using a repeated measures linear model demonstrated that tumor sizes were significantly smaller in mice with macrophage depletion at 9, 12 and 14 days (Figure 2B). At study end, vossicles were collected and flow cytometric analysis showed that tumors had reduced numbers of c-FMS+F4/80+ macrophages with AP treatment (Figure 2C). A trend of smaller tumor weight was observed in mice that survived (Figure 2D). AP treatment in long bones from recipient mice presented significantly fewer macrophages in the bone marrow (c-FMS+F4/80+) and decreased levels of both M2 (c-FMS+CD206+) and M1 (c-FMS+CD86+) macrophages (Figure 2E). This data confirms that macrophage ablation in MAFIA mice efficiently decreased macrophage levels in the vossicle model. To better understand the contribution of macrophages in tumor growth in the absence of bone, subcutaneous tumors were implanted without vossicles in the subcutaneous space of the MAFIA mice, and after 2 weeks tumors were collected and analyzed (Figure 2F). Interestingly, no differences in size and weight were observed after 14 days (Figure 2G), even though macrophage ablation was efficient in subcutaneous tumors (Figure 2H). The Wilcoxon rank test confirmed that the tumor size was not significantly different (*p* = 0.80). Taken together, these data suggest that macrophage ablation in MAFIA mice hinders subcutaneous tumor growth in osseous settings as compared to non-osseous settings. Collectively, these findings suggest that the marrow microenvironment and the macrophages in that environment are particularly vital for tumor growth.

### RM-1 prostate cancer growth in bone of clodronate liposome macrophage-ablated mice

**Figure 3:**
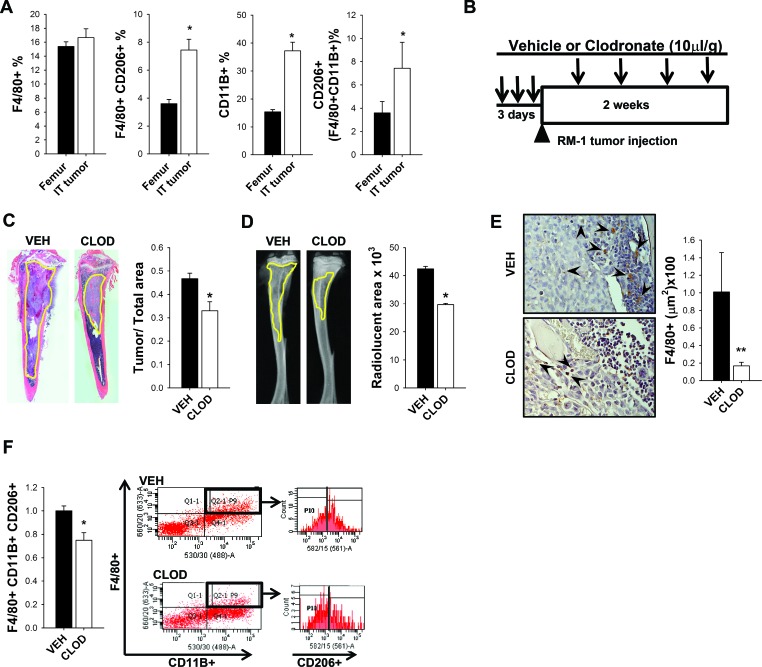
Impact of tumors and clodronate liposomes on macrophages and RM-1 tumor growth in bone **A.** FACS analyses of macrophages (F4/80+), M2 macrophages (F4/80+ CD206+), monocytes (CD11B+) and CD206+ cells gated on F4/80+CD11B+ population in femurs without tumors versus tibiae with intratibial RM-1 tumors in C57Bl6 male mice after 14 days with no macrophage depletion regime. **B.** Experimental design for macrophage depletion regimen and intratibial tumor growth for 2 weeks. Four-six week old C57Bl6 male mice were treated with 3 consecutive injections of clodronate loaded liposomes (CLOD) (10μl/g) or vehicle (VEH). RM-1 murine prostate cancer cells were injected intratibially on the fourth day and booster injections were given every third day for depletion maintenance. Fourteen days later mice were euthanized and evaluated. **C.** and **D.** Histological and radiographic analyses of intratibial RM-1 tumors in vehicle (VEH) and clodronate (CLOD) treated mice. **C.** Representative image of intratibial RM-1 tumor (yellow) and tumor area/tibia area quantification. **D.** Representative radiographic image of tibia and radiolucent area (yellow) quantification (mm^3^). **E.** F4/80 staining (red-brown) on tibial sections (20X) and quantification. Arrows show F4/80+ cells. **F.** Flow cytometric analysis and representative plot of the intratibial RM-1 tumors for M2 macrophages (F4/80+ CD11B+ CD206+). Data for figures A, C, D and F are mean ± SEM; *n* = 18 per group. Data for figure E is mean ± SEM; *n* 12-13 per group. Statistically significant differences were calculated using unpaired *t* tests between treatments. *P < 0.05, **P < 0.01 *vs*. VEH.

The MAFIA mouse model targets c-FMS+ immature macrophages and resulted not only in reduced bone volume but also in decreased tumor growth. To investigate whether the presence of tumors in bone would change the bone marrow population, macrophage populations in femurs without tumors were compared to tibiae containing RM-1 tumors in C57Bl/6J wild type mice. Interestingly, the presence of intratibial tumors significantly boosted the macrophage population in the bones when compared to tumor-free femurs (Figure 3A). Nearly a 2-fold increase in M2 macrophages (F4/80+CD206+), monocytes (CD11B+), and CD206+ expression in F4/80+CD11B+ immature macrophages was observed when tumors were present. To better understand the effect of macrophage depletion at different stages of macrophage maturation, an alternate approach was taken utilizing clodronate loaded liposomes to induce apoptosis of mature phagocytic macrophages. Clodronate liposomes were administered to 4 week old immunocompetent C57Bl/6J male mice for 3 days to initiate depletion. RM-1 prostate cancer cells were then injected intratibially on the fourth day, and booster injections of clodronate were given every three days to maintain macrophage depletion for 2 weeks (Figure 3B). Consistent with the MAFIA mouse model, clodronate treated mice presented smaller intratibial tumors confirmed by histologic and radiographic analyses of tumor area and radiolucent area quantification (Figures 3C and 3D). Efficient macrophage ablation was confirmed by F4/80+ cell staining of tibiae sections (Figure 3E). Figure 3F shows that macrophage ablation by clodronate liposome treatment significantly decreased M2 macrophage F4/80+CD11B+CD206+ cells in intratibial tumors. In summary, targeting the mature phagocytic M2 macrophages resulted in smaller tumors in bone and confirmed the macrophage contribution to tumor growth in bone.

### Clodronate liposome macrophage ablation resulted in increased bone volume

**Figure 4:**
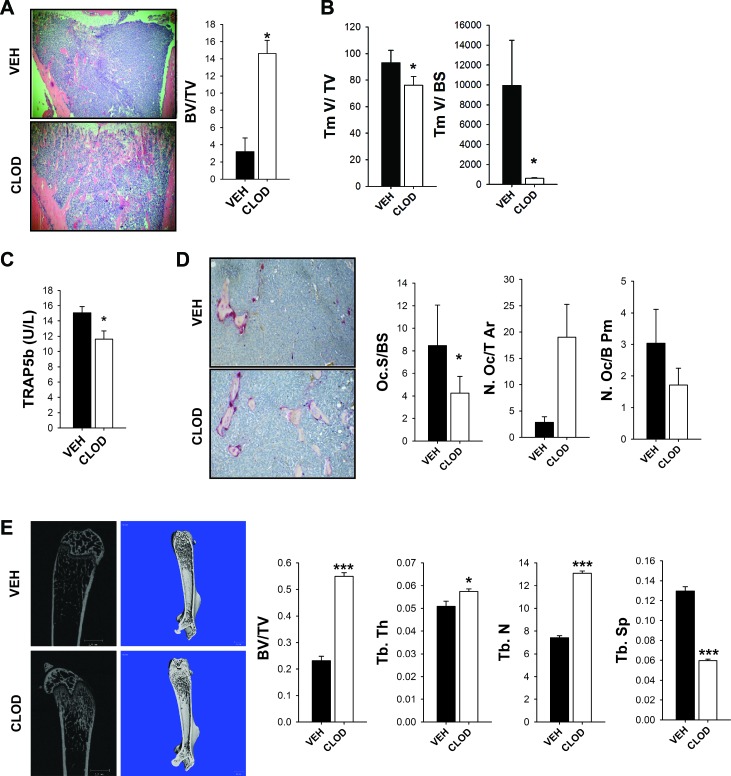
Clodronate liposome treated mice had increased bone volume in an intratibial tumor model *in vivo* Four-six week old C57Bl6 male mice were treated with 3 consecutive injections of clodronate loaded liposomes (CLOD) (10μl/g) or vehicle (VEH). RM-1 murine prostate cancer cells were injected intratibially on the fourth day and booster injections were given every third day for depletion maintenance. Fourteen days later mice were euthanized and evaluated. **A.** Representative image (4X) of intratibial RM-1 tumor of C57Bl6 mice treated with vehicle (VEH) or clodronate (CLOD) and quantification of total bone volume (BV/TV) by histomorphometric analyses at day 14. **B.** Total tumor volume (Tm V/TV) and tumor volume per bone surface (Tm V/BS) were quantified by histomorphometric analyses in intratibial tumors of C57Bl6 mice treated with vehicle (VEH) or clodronate (CLOD) at day 14. **C.** Serum analyses of TRAP5b (units per liter) in C57Bl6 mice with intratibial RM-1 tumors treated with vehicle (VEH) or clodronate (CLOD) after 14 days. **D.** Representative image (20X) of TRAP positive osteoclast surface per bone surface (Oc.S/BS), osteoclast numbers per total tissue area (N.Oc/T.Ar) (*P* = 0.052), and osteoclast numbers per bone perimeter (N.Oc/B. Pm) were quantified by histomorphometric analyses in intratibial tumors of C57Bl6 mice treated with vehicle (VEH) or clodronate (CLOD) at day 14. **E.** Representative 2D and 3D μCT images of femurs without tumors from mice with intratibial tumors show increased trabecular bone volume in clodronate-treated mice. Trabecular total bone volume (BV/TV), trabecular thickness (Tb.Th), number (Tb.N) and spacing (Tb.Sp) were quantified. Data for figures A, B and D are mean ± SEM; *n* = 10-13 per group. Data for figures C is mean ± SEM; *n* = 18 per group. Data for figure E is mean ± SEM; *n* = 7-10 per group. Statistically significant differences were calculated using unpaired t tests between treatments. *P < 0.05, ***P < 0.001 *vs*. VEH.

Both macrophage ablation strategies, the specific transgenic macrophage MAFIA mice, and the pharmaceutical approach targeting the more mature macrophages with clodronate liposome treatment, resulted in smaller RM-1 tumors in bone. To better understand the contribution of mature phagocytic macrophages in bone remodeling and how this may influence skeletal tumor growth in clodronate-treated mice, bone histomorphometric analyses were performed. In contrast to the MAFIA model, clodronate liposome treatment resulted in increased total bone volume (Figure 4A) even though RM-1 tumors were significantly smaller (Figure 4B). Short term macrophage ablation for 2 weeks also resulted in decreased serum TRAP5b levels (Figure 4C). Osteoclast TRAP+ staining showed a significant reduction in the osteoclast surface per bone surface (Oc.S/BS) but no significant changes in osteoclast number per total tissue area (N.Oc/T.Ar) or bone perimeter (N.Oc/B.Pm) (Figure 4D).

To determine whether differences in tumors were due to increased bone volume in macrophage depleted mice, which could have a protective effect, mice were treated for 3 days for initial macrophage depletion and bones were analyzed by μCT (Supplemental Figure 3). No changes were seen in bone volume of trabecular and cortical bone, confirming that bones had similar content at the time of tumor inoculation (Supplemental Figure 3A and 3B).

To exclude the possibility that differences seen in bone volume could be due to the osteolytic nature of RM-1 murine tumor cells, μCT analysis was performed in non-tumor containing femurs (Figure 4E). Consistent with previous studies performed in mice without tumors [20], clodronate liposome induced-macrophage ablation resulted in increased total bone (BV/TV), trabecular thickness (Tb.Th), trabecular number (Tb.N) and reduction in trabecular spacing (Tb.Sp). These data show that targeting the mature macrophage population results in a differential response on bone versus the MAFIA model. Importantly, both macrophage ablation models presented decreased tumor sizes in bone but had different responses in bone remodeling, suggesting that the macrophage contribution to tumor growth is independent of its function in bone remodeling.

### PC-3 human prostate cancer growth in bone of clodronate liposome macrophage-ablated mice

**Figure 5:**
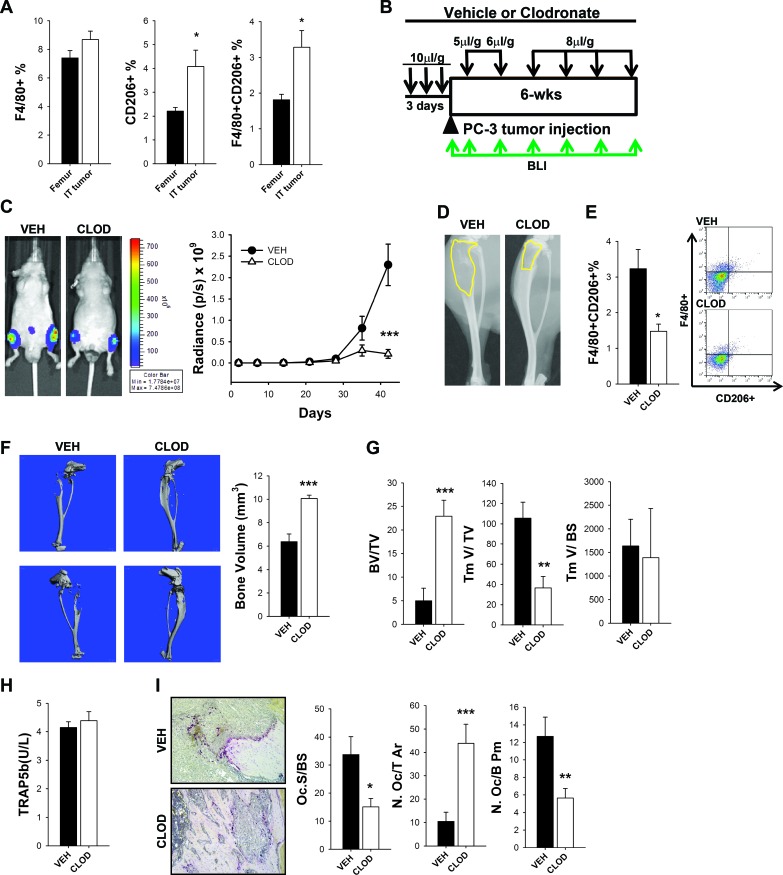
M2 macrophages were increased and contributed to intraosseous tumor growth in human PC-3 intratibial tumors in athymic mice **A.** FACS analyses of bone marrow flush of femurs with no tumors (from mice with intratibial tumors) and tibiae with PC-3 tumors. Macrophages (F4/80+), CD206+ cells and double positive M2 macrophages (F4/80+ CD206+) in athymic mice after 6 weeks were enumerated as a percent of the total cells. **B.** Experimental design of macrophage depletion regimen and intratibial PC-3 tumor growth for 6 weeks. Four-six week old athymic male mice were treated with 3 consecutive injections of clodronate loaded liposomes (CLOD) (10μl/g) or vehicle (VEH). PC-3 luciferase labeled human prostate cancer cells were injected intratibially on the fourth day and booster clodronate or vehicle injections were given every week for depletion maintenance at different doses (5, 6 and 8μl/g) as indicated in figure. Tumor growth was monitored by weekly *in vivo* bioluminescence imaging (BLI) for 6 weeks (green arrows). **C.** BLI representative images at 42 days of intratibial PC-3 tumor growth in vehicle (VEH) and clodronate (CLOD) treated mice. Graph represents BLI measurements for 6 weeks, *n* = 15-17 per group. Statistical analysis for tumor growth was performed by repeated measures linear model, ***P < 0.001. **D.** Representative radiographic image of intratibial PC-3 tumors in athymic mice treated with vehicle (VEH) and clodronate (CLOD) for 6 weeks. Radiolucent area is delineated in yellow. **E.** Flow cytometric analysis of intratibial PC-3 tumors in athymic mice treated with vehicle (VEH) and clodronate (CLOD) for 6 weeks. M2 macrophages (F4/80+/CD206+) quantification of total cells (%) and representative dot plots. **F.** μCT analyses of bones in long term depleted mice with intratibial tumors and representative 3D images shows higher total bone volume (BV/TV) in clodronate treated mice. **G.** Bone histomorphometric analyses in clodronate or vehicle treated mice in intratibial tumor sections of total bone volume (BV/TV), total tumor volume (TmV/TV) and tumor volume per bone surface (TmV/BS). **H.** Serum analyses of TRAP5b (units per liter) in athymic mice with intratibial PC-3 tumors treated with vehicle (VEH) or clodronate (CLOD) after 42 days. **I.** Bone histomorphometric analyses of intratibial PC-3 tumors in athymic mice treated with vehicle (VEH) or clodronate (CLOD) for 42 days. Representative images (10X) of TRAP positive osteoclasts stained in red. Osteoclast surface per bone surface (Oc.S/BS), osteoclast number per total tissue area (N.Oc/T.Ar), and osteoclast number per bone perimeter (N.Oc/B.Pm) were quantified. Data for figure A is mean ± SEM; *n* = 10-15 per group. Data for figures E, F and H are mean ± SEM, *n* = 15-19 per group, Data for figures G and I are mean ± SEM, *n* = 14-15 per group. Statistically significant differences were calculated using unpaired t tests between treatments. *p < 0.05, **p < 0.01, ***p < 0.001.

A human prostate cancer model was employed to further confirm the macrophage contribution in skeletal tumor growth using PC-3 cells in the intratibial tumor inoculation experiment, this time using athymic mice. To delineate macrophage involvement in bone tumors, bone marrow populations were compared between the bones with intratibial tumors and non-tumor femurs of mice treated with clodronate or vehicle control. Significant increase of M2 macrophage (F4/80+ CD206+) cells was found in the presence of tumors, consistent with the contribution of M2 macrophages in skeletal tumor growth (Figure 5A).

Three days after initial depletion with vehicle control or clodronate liposome treatment, luciferase tagged PC-3 cells were injected in the tibia and tumors were monitored by bioluminescent imaging every week for 6 weeks. In preliminary experiments, high dosage of clodronate booster injections (10μl/g) resulted in debilitated mice. Thus, an optimized, decreased level of booster injection (5-8 μl/g) was given weekly for 6 weeks to maintain macrophage depletion while minimizing adverse effects (Figure 5B). After 6 weeks, bioluminescence imaging showed that clodronate liposome treated mice presented significantly smaller tumors in the tibia compared to controls, confirming a role of macrophages in PC-3 tumor growth in bone (Figure 5C). Tibiae with tumors were collected (Figure 5D) and flow cytometry confirmed significant ablation of M2 macrophage (F4/80+CD206+) cells (Figure 5E and Supplemental Figure 4).

In addition, μCT and histomorphometric analyses of tibiae demonstrated that clodronate liposome treatment increased bone volume and resulted in smaller tumors (Tm/TV) (Figures 5F and 5G). Serum TRAP5b was not changed after 6 weeks of macrophage ablation (Figure 5H). TRAP staining showed significant reductions in osteoclast surface per bone surface (Oc.S/BS), number of osteoclasts per bone perimeter (N.Oc/B. Pm), and increased total numbers of osteoclasts (N.Oc/T.Ar) (Figure 5I). Similar to RM-1 prostate cancer cells, these data suggest that macrophages contribute to PC-3 prostate cancer growth in bone independent of the bone response.

## DISCUSSION

Macrophages are heterogeneous myeloid lineage cells that comprise diverse functions towards the immune defenses [7, 24]. They rapidly respond to stimulants of injury, infection and other changes in the environment, having a diverse differentiation and activation profile according to the stimuli received [4]. Simplistically, macrophages can be described as classically activated (also known as M1) or alternatively activated (known as M2). M1 macrophages respond to infection and generate pro-inflammatory responses to exert their role as cell killers against invaders and pathogens. On the other hand, M2 macrophages participate in wound healing, tissue repair, and moderate inflammatory responses as anti-inflammatory cells. In the context of tumors, M2 macrophages are also termed tumor-associated macrophages (TAMs) because of the similar immunosuppressive roles they have, contributing to tumor growth, progression and metastasis of different types of cancer [5, 25, 26]. In bone, ‘osteomacs’ are the resident macrophages that line the periosteal and endosteal tissues and have essential roles, supporting osteoblast maintenance and functional activity [17]. Moreover, these resident macrophages impact bone healing, remodeling and hematopoietic stem cell maintenance [3, 18-20]. However, how resident macrophages and their impact in the bone microenvironment affect skeletal tumor growth is unclear.

The present study demonstrated that regardless of the strategy used, targeting macrophages for depletion hindered tumor growth in bone. In the transgenic MAFIA mouse, intratibial tumors were smaller in macrophage depleted mice compared to controls. Depletion of macrophages in the bone marrow was confirmed by flow cytometry looking at very specific macrophage markers (Gr1^lo^, F4/80^+^, c-FMS^int^ and CD11b^hi^) prior to tumor inoculation. When subcutaneous tumor growth was analyzed after 2 weeks of inoculation, no difference was observed, even though there was a significant reduction in macrophage c-FMS+F4/80+ numbers in both subcutaneous tumors and bone marrow. Interestingly, there was a significant difference in tumor size in the vossicle model at early time points in macrophage depleted mice. We hypothesized that when tumors grew within the bone, a reduction in macrophages impacted tumor growth, but when tumors outgrew the bone area the macrophages were no longer the driving factor. This suggests that macrophage depletion yields greater impact in the myeloid cell-rich bone marrow compartment, which is the case in the intratibial tumor model. To further validate the role of macrophages in skeletal tumor growth, a more narrowly focused macrophage depletion model was introduced with clodronate liposome treatment. Mature macrophage depletion was efficient, and similar to the MAFIA mouse model, there were smaller tumors in macrophage depleted mice. In this model, tibiae with tumors and tumor-free femurs were both analyzed by FACS for macrophage markers. Interestingly, the presence of tumors in tibiae significantly increased M2 macrophages (F4/80+ CD206+) and clodronate-induced depletion significantly reduced M2 macrophages within the intratibial tumors. Similar results were also confirmed when the human PC-3 prostate cancer cell line was inoculated in the tibiae of athymic mice over 6 weeks. These data not only confirmed the contribution of macrophages in tumor growth, but also demonstrated that tumor development in bone increased M2-like macrophages, which emphasizes the vital role of macrophage polarization in tumor progression.

Altering the bone microenvironment, rich in myeloid cells, prior to tumor inoculation affected tumor growth in bone. Park *et al*. demonstrated that a transient expansion of myeloid cells from a single dose of cyclophosphamide prior to intracardiac tumor inoculation of prostate cancer cells increased localization and growth in bone [10]. Moreover, tumor derived factors such as PTHrP (parathyroid hormone-related protein) and CCL2 induce direct and indirect changes in the bone and tumor microenvironment that contributed to tumor growth [10-15, 27-31]. For example, PTHrP derived from solid prostate tumors, primed and recruited myeloid cells, also known as myeloid derived suppressor cells (MDSCs) which contributed to solid tumor growth. CCL2, a contributing factor for M2 macrophage polarization, is a chemokine also known for its role in both tumor growth and myeloid cell modulation, [10-15, 28-30]. Specifically, in bone, CCL2 is known to be involved in a destructive cascade, being released after PTHrP stimulation of osteoblasts, which in turn secretes CCL2 in the bone marrow activating osteoclastogenesis and tumor growth [28].

Osteoclasts are key players in skeletal metastasis, and are specialized cells differentiated from myeloid cells. A particular challenge in bone, when studying macrophages is the effects that macrophage depletion may have on osteoclast activity. Previous research has shown that macrophage depletion in MAFIA mice resulted in decreased bone remodeling [17, 20]. We have previously demonstrated that macrophage depletion in 16 week old MAFIA mice resulted in reduced serum TRAP5b levels after 2 weeks of depletion, but that no difference was observed after 4 and 6 weeks of depletion [20]. P1NP levels reflecting bone formation activity were reduced at 2 and 4 weeks after macrophage depletion, and at 6 weeks, trabecular and cortical bone volume were significantly decreased in macrophage depleted mice. Therefore, in the current study, it was expected that macrophage depletion in MAFIA mice would result in decreased bone remodeling even in the tumor context. However, no difference was observed in local osteoclast numbers when tumors were present, although systemic osteoclast TRAP5b levels were reduced after 2 weeks of macrophage depletion in MAFIA mice. This suggests that the local presence of tumors preferentially preserved osteoclast activity while osteoclasts may be reduced in more quiescent sites. Limitations in bone histomorphometric analysis of MAFIA mouse model in the presence of tumors are acknowledged, since both macrophage depletion and the presence of larger tumors in the vehicle treated group resulted in reduced trabecular bone and could influence osteoclast quantification. Clodronate liposome administration for 2 weeks also resulted in reduced TRAP5b levels. A longer macrophage depletion regimen for 6 weeks resulted in reduced osteoclast numbers per bone perimeter and osteoclast surface per bone surface, but differences were not seen in TRAP5b levels. This may be a reflection of increased bone content while the osteoclast activity is stabilized as well as a differential response in the local environment versus the systemic circulation. Many tumor derived factors are known to promote osteoclastogenesis such as PTHrP and CCL2 [10-12, 28, 31, 32]. Still, the effect of macrophage depletion on osteoclasts cannot be fully determined since they share the same precursors. Indeed, in the same way these different macrophage depletion strategies may affect osteoclasts, current osteoclast therapies, such as bisphosphonates, may significantly affect macrophage populations. The current study addresses the understudied question: To what extent do macrophages play a role, not only in the bone response, but also in tumor growth in the skeletal context? Overall, these data suggest that even though macrophage depletion may suppress in part osteoclast differentiation/activation, tumors compensate for the suppression, driving the differentiation/activation of available macrophage progenitors towards osteoclasts.

An interesting finding in the present study was that tumors were consistently smaller in macrophage depleted mice, even though paradoxical results of macrophage depletion on the bone were observed. Targeting early progenitor cells under the c-FMS promoter in the transgenic MAFIA mouse model resulted not only in reduced tumor growth, but also in decreased bone volume in the hind limbs of macrophage depleted mice. Previous studies reported the role of macrophages in the regulation of osteoblast function *in vivo* [17]. It was demonstrated that osteal tissue macrophages lie in close association with osteoblasts and contribute to osteoblast mineralization [17]. Our findings suggest that differences in bone volume observed with AP-treatment in MAFIA mice were not due to direct effects on osteoblast function or osteoclast number, but were via its impact on osteoclast activity. In the bone tumor microenvironment the osteolytic activity of cancer cells likely prevails, reflected by the lack of differences in the bone volume between VEH and AP (smaller tumors) groups. As previously reported, macrophages contributed to bone-remodeling functions as seen in the MAFIA model [17]. Depletion of macrophages *in vitro* with a single treatment, followed by the analysis of its effect in osteoblast differentiation, showed that not only was there no diminution of the effect, but, in fact, the opposite occurred. This, as well as the entirety of experiments, strongly suggests against any osteoblast specific effect. Still, we acknowledge that it is not impossible that some small effect could exist. In contrast, targeting mature phagocytic macrophages with administration of clodronate liposomes resulted in increased bone volume, while tumor growth was still hindered in macrophage depleted mice in both murine RM-1 and human PC-3 prostate cancer cell models. These results suggest that the effect of macrophage depletion on tumor growth was independent of its effect on bone tissue. We have previously demonstrated that osteal macrophages support bone remodeling and PTH anabolic actions in the adult murine skeletal system [20]. Clodronate loaded liposomes triggered an opposite effect resulting in an osteogenic microenvironment and augmented PTH anabolic actions. The main difference observed was that the phagocytic myeloid CD68+ cells that include macrophages were differential according to the depletion model. In clodronate liposome treated mice there was a significant increase in CD68+ cells which led to the hypothesis that selective depletion of phagocytic macrophages activates a compensatory expansion and activation of the mononuclear phagocytic system for cell clearance of apoptotic cells and debris.

Apoptotic cell clearance through phagocytosis, also known as efferocytosis, has been shown to contribute to tumor growth and modulation of immune responses [33-35]. For example, macrophage efferocytosis of apoptotic tumor cells mediated through milk fat globule EGF-8 (MFG-E8), a protein that facilitates this process, modulates macrophage polarization into M2 tumor associated macrophages [36]. This may be a mechanism by which macrophages play a role not only in tumor growth and polarization, but also in bone responses. How phagocytic macrophages and the mechanisms utilized for cell clearance may influence tumor growth is an intriguing avenue of future investigation.

In summary, this study shows strong evidence that resident macrophages contribute to prostate cancer growth in bone. The presence of tumors was sufficient to increase tumor associated macrophages in the bone marrow. Moreover, targeting earlier progenitor cells or mature phagocytic macrophages had a differential effect on bone tissue, but both strategies hindered tumor growth in bone. The MAFIA mouse model that targets immature macrophage progenitors displayed reduced bone. On the other hand, clodronate liposome treatment targeted mature phagocytic macrophages and resulted in increased bone volume. In conclusion, these data support that macrophages have important roles in tumor growth in bone. Moreover, macrophage depletion hindered tumor growth independently of the paradoxical effect on bone content, suggesting that macrophages in bone may be more important to tumor growth than the bone itself.

## MATERIALS AND METHODS

### Animals and cell lines

All animal experiments were performed with the approval of the University of Michigan Committee for the Use and Care of Animals. MAFIA mice and control wild type mice (C57BL/6J) were purchased from Jackson Laboratories (Bar Harbor, ME). Male athymic mice were purchased from Harlan Laboratories.

PC-3 cells were initially purchased from ATCC, and subsequently labeled with luciferase as previously described [37]. RM-1 cells were originally obtained from Dr. Timothy C. Thompson (Baylor College of Medicine, Houston, TX). Both cell lines were commercially authenticated by IDEXX Bioresearch (Westbrook, ME).

### MAFIA mouse model with intratibial RM-1 tumors

The macrophage Fas-induced apoptosis (MAFIA) C57BL/6-Tg mouse has a c-FMS promoter (CSF-1 receptor) expressed in phagocytic cells such as macrophages, and to a lesser extent in dendritic cells. Administration of a synthetic dimerizer, AP20187 (Clontech Laboratories, Inc.) induces Fas-mediated apoptosis in resting and cycling myeloid lineage cells. MAFIA mice (12-14 week old) received 3 daily i.v. injections of AP20187 (10 mg/kg body weight) or vehicle for macrophage depletion as previously described [20]. Booster i.p. injections (1mg/kg) were administered every 3 days to sustain macrophage suppression. After initial depletion, RM-1 murine metastatic prostate cancer cells were inoculated via intra-tibial injection under anesthesia as previously described [38]. Upon necropsy, bones were analyzed histomorphometrically to evaluate tumor, bone area and resorption indices. Serum TRAP5b was measured as an indicator of bone remodeling activity.

### Intratibial tumors

After initial macrophage depletion, RM-1 murine prostate cancer cells (2×10^3^ cells in 20 μl HBSS) or luciferase tagged PC-3 human prostate cancer cells (2×10^4^ cells in 20 μl of HBSS) were inoculated via intra tibial injection under anesthesia as previously described [38]. To determine changes in the bone microenvironment, both legs were injected for tumor growth and subsequent analyses were performed. Upon necropsy, one limb was dissected and bone marrow was flushed with PBS for FACS analyses. The other limb was immediately fixed in 4% paraformaldehyde for 24 hours and maintained in 70% ethanol for further *ex-vivo* analyses.

X-ray images were taken using the Faxitron (Faxitron ^®^) at 35kV for 45 seconds and intratibial tumors were analyzed for radiolucency. Radiolucent areas were delineated utilizing the ImageJ program for total area determination. Femurs were utilized as controls for differences of treatment effect on bone radiolucency. Blinded measurements were performed by two different investigators and averaged for each sample. Tumor presence was confirmed in histological sections stained with H&E as previously described [22]. Images were taken at 10x and stitched together to construct whole bone images. Histological tumor areas were delineated utilizing the ImageJ program for total area determination relative to total bone area.

### Vossicle (vertebral body) subcutaneous RM-1 tumors

Four day old MAFIA mice received 3 daily i.p. injections of AP20187 (10 mg/kg body weight) or vehicle for macrophage depletion. Lumbar vertebrae were dissected and sectioned into single vertebral bodies (vossicles) as previously described [23]. Subsequently, two thousand RM-1 tumor cells were injected into the vossicle and implanted in the backs of (12-14 week old) MAFIA mice that had been previously treated with AP20187 or vehicle for 3 days. A total of four vossicles with tumor cells were implanted in each mouse and 14 days later harvested for analyses.

For subcutaneous tumors, one thousand RM-1 tumor cells were suspended in 100 μL Hank's balanced salt solution and 1:1 mixed with growth factor-reduced Matrigel (BD Biosciences). Mixed cells were then injected subcutaneously in the back of macrophage depleted or vehicle treated MAFIA mice. A total of 2 subcutaneous tumors were implanted in each mouse and 14 days later harvested for analyses.

Tumors were measured by calipers every 3 days and tumor volume was calculated using the formula: Volume = ½ x *a* x *b*^2^, where *a* is the long diameter and *b* is the short diameter.

### Clodronate loaded liposomes

Clodronate (dichloromethylene bisphosphonate)-loaded liposomes (clodronate liposomes) or control PBS-loaded liposomes (PBS liposomes) were purchased from ClodronateLiposomes.com (The Netherlands). Four-week-old male athymic or C57BL/6 mice were treated with clodronate liposomes (10 μl/g body weight) for 3 consecutive injections, then given every third day for 2 weeks (C57BL/6) or every week at a reduced dosage ( from 5 μl/g; athymic mice) for the next 3 weeks.

Different concentrations of clodronate for the booster injections were given based on a preliminary experiment in athymic mice, which determined the optimal dosage to keep macrophages depleted and avoid lethality. Briefly, in a preliminary experiment it was observed that after the 3 day initial depletion and intratibial injection, mice were severely affected by the first booster injection (10 μl/g). Therefore, a significant reduction in the clodronate amount for the first booster injection was administered (5 μl/g) and increased dosages for the later booster injections (6 μl/g and 8 μl/g), for macrophage depletion maintenance for up to 6 weeks.

After initial depletion, RM-1 or luciferase tagged PC-3 cells were injected in the tibia of mice. *In vivo* bioluminescence imaging (BLI) was performed every 3 days to monitor PC-3 ^Luc^ tumor growth.

### Flow cytometric analyses

Immediately after sacrifice, bone marrow cells were flushed with FACS buffer (PBS supplemented with 2% FBS and 2 mM EDTA) and 1 × 10^6^ cells were incubated with fluorescent-conjugated antibodies. Antibodies for flow cytometric analyses included: anti-mouse F4/80 FITC (Abcam, Clone A3-1), F4/80 APC (Biolegend, Clone A3-1), anti-mouse CD11B APC (eBioscience, Clone M1/70), CD206 PE (AbD Serotec, Clone MR5D3), anti-mouse GR-1 PEcy3 (BDBiosciences, Clone RB6-8C5) and CD86 PE (BDBiosciences, Clone GL1). C-FMS+ cells were identified by GFP expressed in the MAFIA mice. Matched isotype controls were used and pre-gating was performed excluding dead cells and debris in SCC x FSC graph and aggregates in FSC-W x FSC-H and SSC-W x SSC-H.

For macrophage analysis in tumor tissue, tumors were mechanically dissociated, followed by digestion in complete RPMI-1640 media supplemented with type I collagenase (5 mg/mL; Sigma-Aldrich), 0.5% FBS and 1% Penicillin-streptomycin for 4 hours. Cells were washed in PBS multiple times to eliminate collagenase and passed through 75μm cell strainer for single cell suspension. Viable cells were counted and 1 × 10^6^ cells incubated in FACs staining buffer containing combinations of antibodies or isotype controls. After washing, cells were analyzed on BD FACSAria™ III.

### Histology and bone histomorphometric analyses

Tibiae were fixed in 4% paraformaldehyde solution (24 hours) and decalcified in 10% EDTA (21 days) at 4°C. Paraffin-embedded tibial sections were stained with tartrate resistant acid phosphatase (TRAP) staining assay system (Sigma-Aldrich) per manufacturer's instructions. Histomorphometric analyses were performed according to standard protocols [39]. Briefly, 4 fields in 5μm sections were analyzed at 20x magnification in areas 250μm below the lowest point of the growth plate using Osteomeasure software (OsteoMetrics Inc. Atlanta, GA). Within the regions analyzed, tumors were delineated and quantified as tumor volumes (TmV/TV) and (Tm V/BS).

### μCT imaging

Fixed tibiae and femurs were analyzed by μCT imaging (eXplore Locus, GE Healthcare, London, ON, Canada; Scanco μCT-100, Medical AG, Bruttisellen, Switzerland). Bones were fixed in formalin and embedded in 1% agarose. After placing them in a 19 mm diameter tube, bones were scanned over their entire length using a μCT system (μCT100 Scanco Medical). Scan settings were: voxel size 12 μm, medium resolution, 70 kVp, 114 μA, 0.5 mm AL filter, and integration time 500 ms. Trabecular bone parameters were measured over 50 slices using a threshold of 28% at 15 slices distal to the growth plate; cortical bone was measured over 30 slices at 60% of the bone length distal to the tibio-fibular joint (TFJ) using a 28% threshold.

### Immunohistochemical F4/80 staining

Tibiae were fixed in 4% paraformaldehyde/PBS (4°C, 24hr), decalcified in 14% EDTA for 2 weeks, and embedded in paraffin. Immunohistochemical staining was performed, using the Cell & Tissue Staining Kit (HRP-DAB system; R&D systems) and rat monoclonal anti-mouse F4/80 (1:100, Abcam ab6640, Cambridge, UK). Percentages of F4/80+ cells were quantified using NIS Elements software (Nikon). The mean area positive for F4/80+ cells was calculated for each tissue within 4 different areas of the tissue.

### Blood serum biochemical assay

ImmunoAssay kits obtained from IDS were used to measure serum TRAP5b following manufacturer's instructions.

### Differentiation and mineralization of calvarial osteoblasts *in vitro*

Primary calvaria cells were isolated from 3 MAFIA mice (M1, M2 and M3) and propagated for 4 days in 100 mm tissue culture (TC) plates using αMEM media. When cells reached confluency they were reseeded in three TC plates (12 wells) at 50000 cells/cm^2^. Half of the wells [6] were treated with the dimerizer drug AP20187 (Clontech) at a concentration of 250 nM. For differentiation and mineralization, cells were treated either with ascorbic acid alone or ascorbic acid (AA) plus β-glycerol phosphate (BGP) for 12 and 14 days respectively. Culture media was changed every other day. Total RNA was isolated from each well of the AA-treated plate and the relative osteocalcin gene expression was analyzed using the BGLAP3 probe (Cat # Mm03413826_mH; Applied Biosystems). The plate treated with AA plus BGP was subjected to Von Kossa mineralization assay. The quantification of total mineralized area was analyzed using Image J software.

### Statistics

Continuous outcomes were described using mean ± standard error of the mean (SEM). GraphPad Instat statistical program was used to perform unpaired *t*-tests with significance of *p* < 0.05. For uncentered or non-normal data, Wilcoxon rank test was implemented. The tumor size in the vossicle model used the average of the 4 vossicles implanted in each mouse at each time for the outcome. Tumor growth in the mouse models was analyzed using repeated measures mixed models including mouse group, day and the interaction of group and day as independent covariates. To obtain linear data to meet model assumptions, the log of tumor size was modeled but for ease to the reader, graphs present the actual tumor size means with SEMs. Growth over time was tested between groups using an interaction of group and time in the model if the growth was linear. Pairwise group differences were explored using post-hoc tests adjusted for multiple comparisons.

## SUPPLEMENTARY MATERIAL FIGURES


