# Supplementary material for: Bone marrow macrophages support prostate cancer growth in bone
Source: Oncotarget. 2015 Oct 8;6(34):35782–96. doi: 10.18632/oncotarget.6042 (PMC4742141; doi:10.18632/oncotarget.6042)
Supplement: Supplementary file 1 [file oncotarget-06-35782-s001.pdf]

Bone marrow macrophages support prostate cancer growth in bone

Supplementary Material

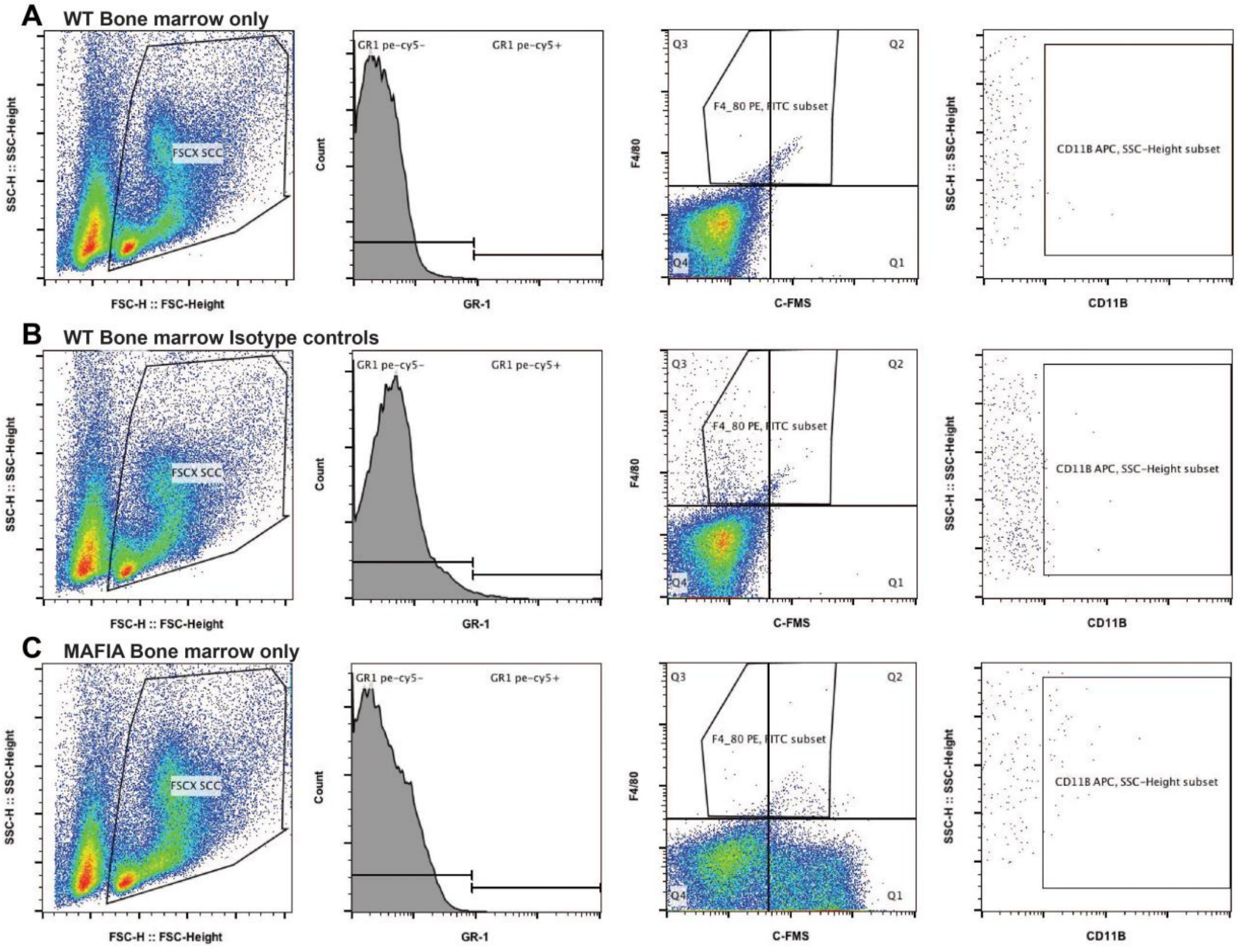

Supplemental Figure 1: Negative controls for FACS of MAFIA mice macrophage depletion

A. Wild type (WT) bone marrow cells were collected and used as negative control.

B. Wild type (WT) bone marrow cells were incubated with isotype negative antibodies.

C. MAFIA bone marrow cells only were used for positive c-FMS+ gating of GFP+ cells.

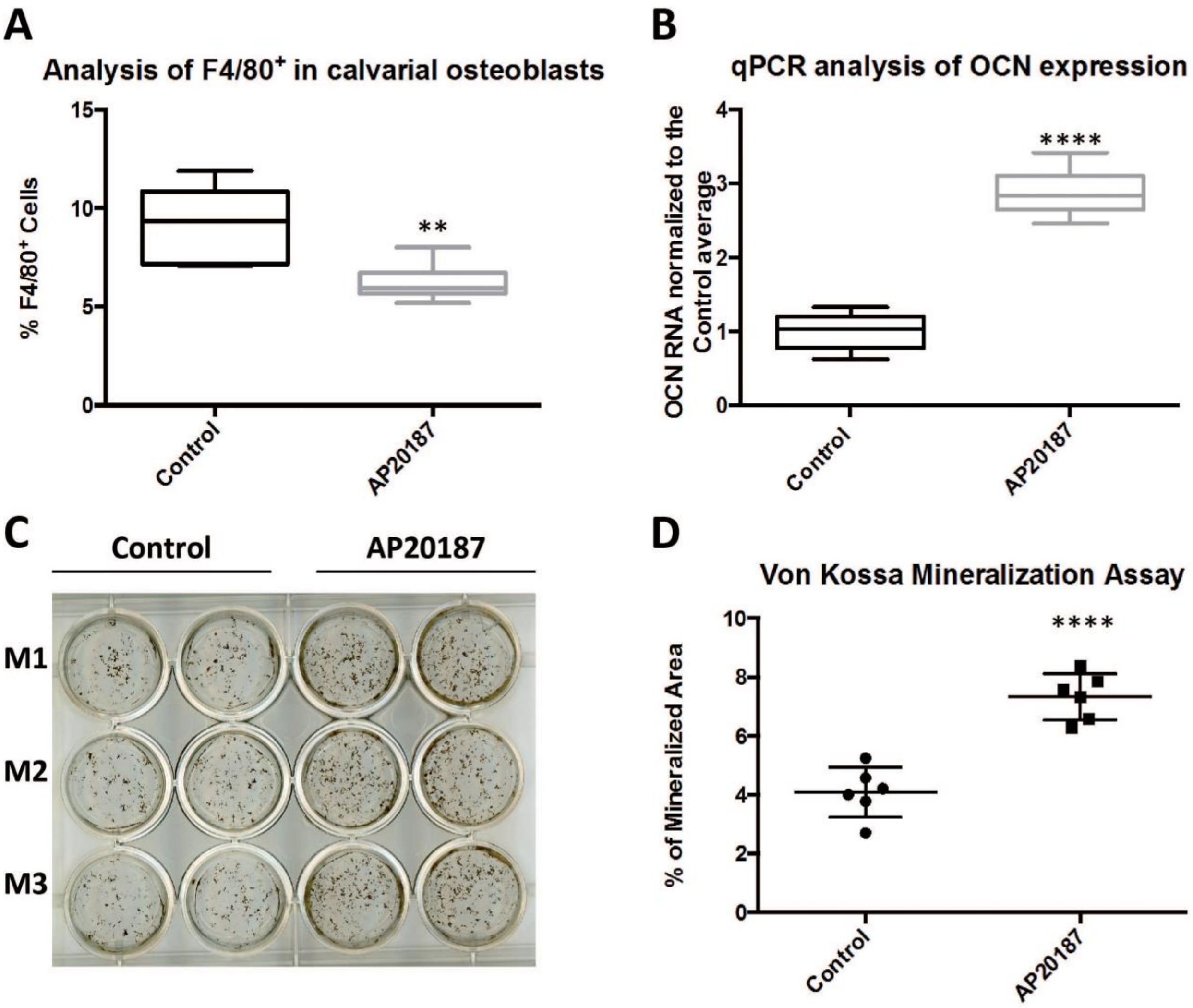

Supplemental Figure 2: AP20187-mediated ablation of F4/80+ cells stimulates differentiation and mineralization of calvarial osteoblasts from MAFIA mice *in vitro*. Primary calvarial cells were isolated from 3 MAFIA mice (M1, M2 and M3) and cultured for 4 days in 100 mm tissue culture (TC) plates using αMEM media. When cells reached confluence they were reseeded in 12 wells plates at 50.000 cells/cm^2. Cells were treated with the dimerizer drug AP20187 (Clontech) at a concentration of 250 nM, and on the next day, F4/80+ macrophages were analyzed by flow cytometry. Cells were then treated with ascorbic acid alone or ascorbic acid (AA) plus β-glycerol phosphate (BGP) for 12 and 14 days respectively for mineralization. The total RNA was isolated from each well of the AA-treated plate and the relative osteocalcin gene expression was analyzed using the BGLAP3 probe (Cat # Mm03413826_mH; Applied Biosystems). (n=3/gp)

A. Flow cytometric analysis of F4/80+ macrophages in MAFIA mice calvarial osteoblasts after AP20187 or VEH control treatment. Macrophages were significantly reduced with AP treatment.

B. Calvarial osteoblasts were treated with ascorbic acid alone or ascorbic acid (AA) for 12 days after AP20187 or VEH control treatment and the total RNA was isolated for osteocalcin gene expression.

C and D. Calvarial osteoblasts were treated with ascorbic acid (AA) plus β-glycerol phosphate for 14 days (BGP) after AP20187 or VEH control treatment and osteoblast mineralization was determined by von Kossa staining

C. Von Kossa staining of mineralized area

D. Staining quantification of total mineralized area .

The experiment was repeated two times showing similar results. Data are mean ± SEM. Statistically significant differences were calculated using unpaired t tests between treatments.

** $p<0.01$, *** $p<0.001$.

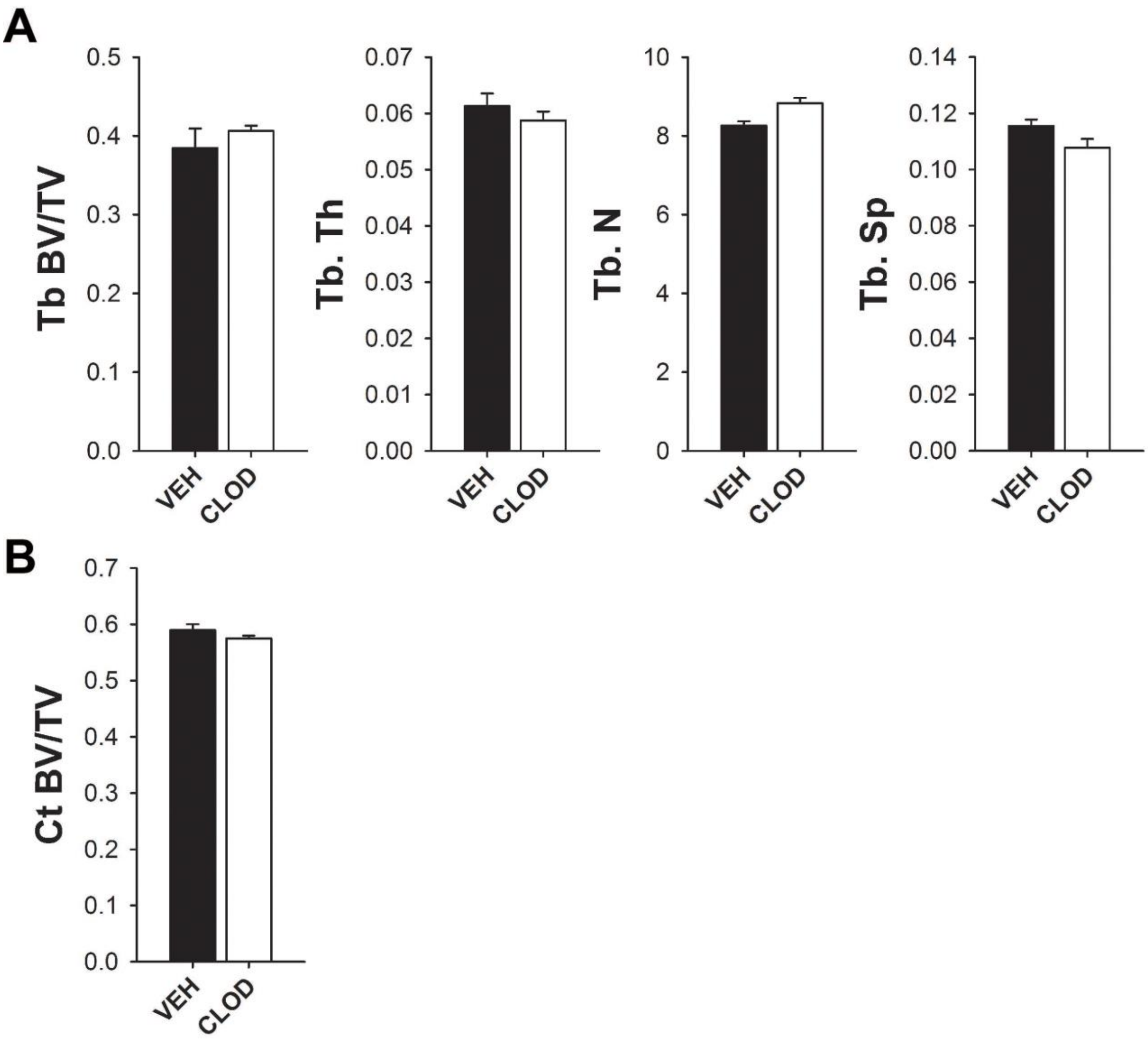

Supplemental Figure 3. Initial macrophage depletion for 3 days by clodronate liposome did not increase bone volume of macrophage depleted mice.

A. μCT images of tumor free tibias that were previously treated with clodronate or vehicle for 3 consecutive days. Data showed no differences in trabecular bone volume (BV/TV), trabecular thickness (Tb.Th), number (Tb.N) and spacing (Tb.Sp)

B. Cortical bone volume (BV/TV). Data are mean ± SEM; n=5 per group.

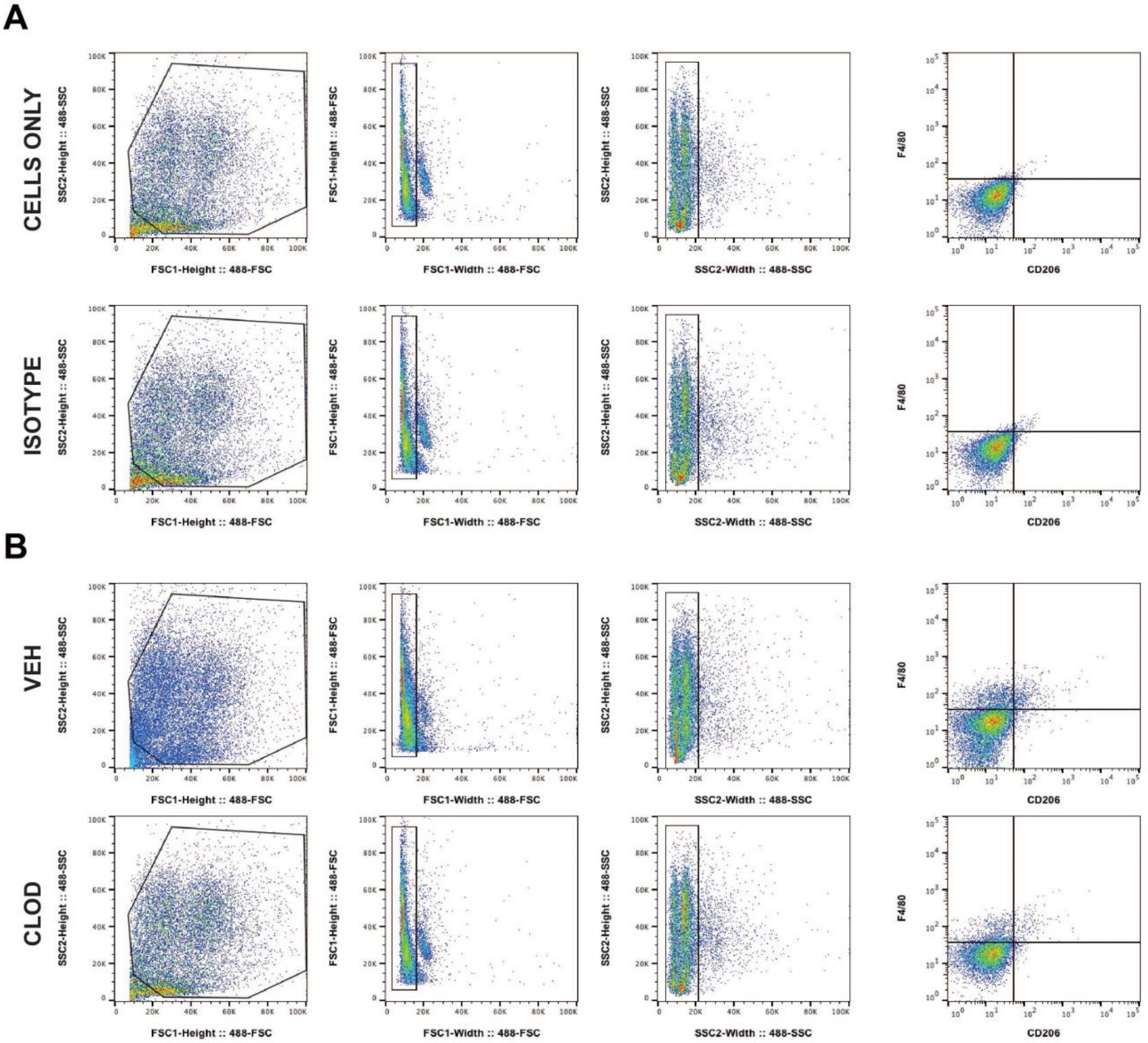

Supplemental Figure 4: Efficient macrophage depletion of athymic mice with intratibial PC-3 tumors after clodronate treatment.

A. Cells were pregated for cell aggregates elimination by FSCx SCC, FSC-H x FSC-W and SSC-H x SSC-W. Unstained bone marrow cells only and bone marrow cells that were incubated with isotype negative antibodies, were used as negative controls.

B. Representative image of Vehicle (VEH) or Clodronate(CLOD) treated group for 2 weeks after tumor inoculation. Data are mean ± SEM; n=15-20 per group.
